# Supplementary material for: Intracortical brain-computer interface for navigation in virtual reality in macaque monkeys
Source: Sci Adv. 2026 Apr 15;12(16):eadw3876. doi: 10.1126/sciadv.adw3876 (PMC13082338; doi:10.1126/sciadv.adw3876)
Supplement: Supplementary file 1 — Supplementary Results Figs. S1 to S6 Tables S1 to S5 Legends for movies S1 to S7 [file sciadv.adw3876_sm.pdf]

Science Advances
AAAS

Supplementary Materials for

Intracortical brain-computer interface for navigation in virtual reality in macaque monkeys

Ophelie Saussus *et al.*

Corresponding author: Peter Janssen, peter.janssen@kuleuven.be

Sci. Adv. **12**, eadw3876 (2026)
DOI: 10.1126/sciadv.adw3876

The PDF file includes:

Supplementary Results
Figs. S1 to S6
Tables S1 to S5
Legends for movies S1 to S7

Other Supplementary Material for this manuscript includes the following:

Movies S1 to S7

Supplementary Results: Task-Specific time to target Comparisons

In this supplementary analysis, we analyzed the time to target across task-relevant conditions using two-sided Mann-Whitney U tests. In the 3D Center-out task, Monkey 2 showed no significant difference in time to target between upper and lower targets ($U = 128357$, $p = 0.16$, $N = 988$ trials), whereas Monkey 3 required significantly more time to reach the upper targets ($U = 58986$, $p = 1.11 \times 10^{-6}$, $N = 630$ trials), highlighting individual differences in adapting to vertical spatial control.

In the Respawn task, both monkeys took significantly longer to reach the target on trials involving a mid-trial target jump (Monkey 2: $U = 142631$, $p = 1.16 \times 10^{-12}$, $N = 960$ trials; Monkey 3: $U = 90120$, $p = 3.24 \times 10^{-10}$, $N = 775$ trials). This increase—less than 330ms for Monkey 2 and 190ms for Monkey 3—is expected due to the need to redirect toward a new target position.

In the Obstacle task, both monkeys exhibited a modest but significant increase in the time to target when obstacles appeared on-path compared to off-path (Monkey 2: $U = 32963$, $p=0.01$, $N = 630$ trials; Monkey 3: $U = 49891$, $p = 0.01$, $N = 738$ trials). Although these differences were statistically significant, the absolute increase in movement time was relatively small ($\leq$ 250ms), suggesting that the monkeys were able to efficiently navigate around obstacles with only minimal impact on performance.

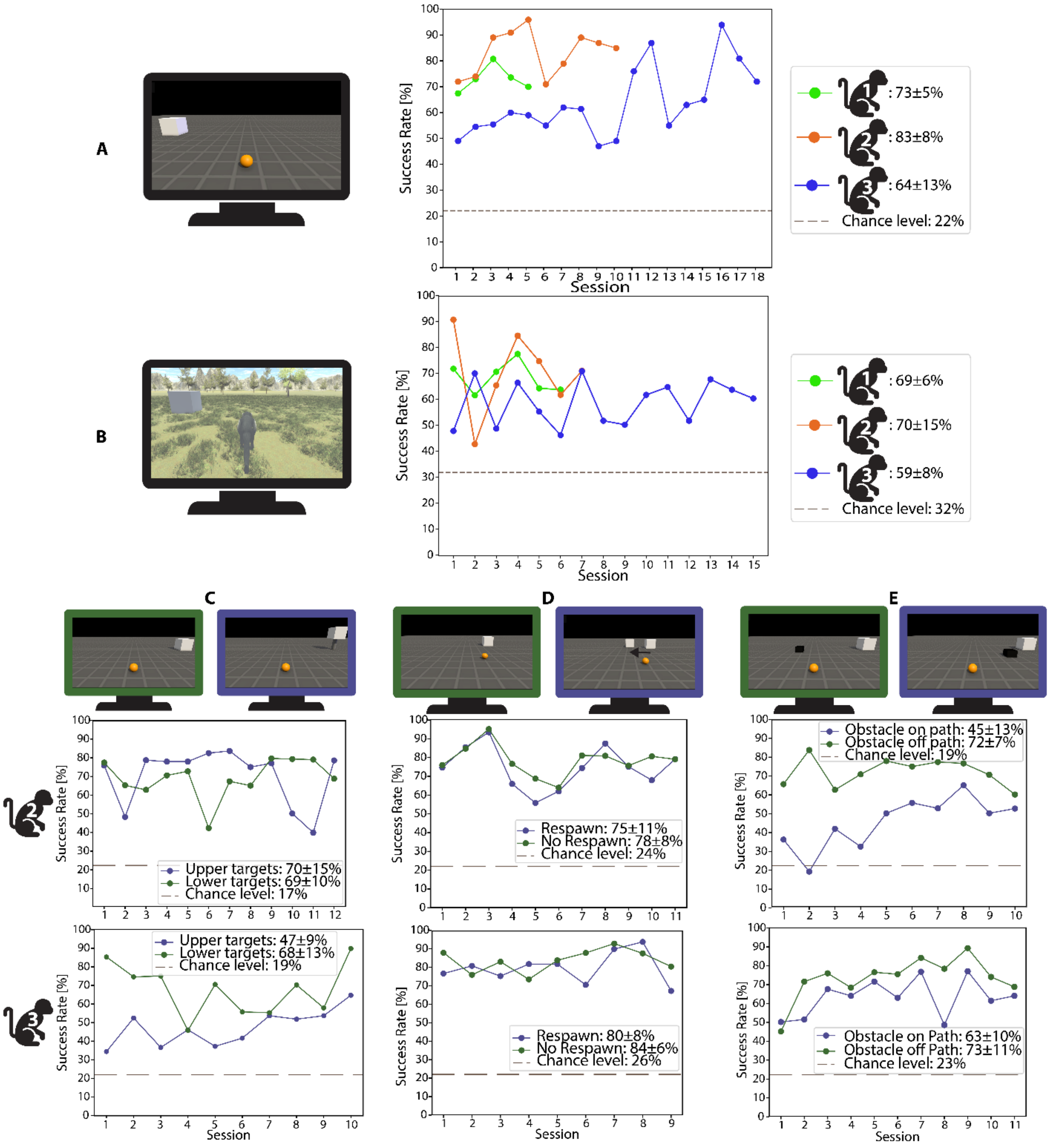

Fig. S1. Success rate over time for different tasks. Success rate of each session, together with the estimated chance level and the average success rate ± standard deviation of all sessions. Success rate of Monkey 1, 2 and 3. **A** Center-out task. **B** Continuous Navigation task. **C** 3D Center-out task: upper targets (blue) and lower targets (green). **D** Respawn task: respawn (blue) and no respawn (green). **E** Obstacle task: trials with obstacle on path (blue) and obstacle off path (green).

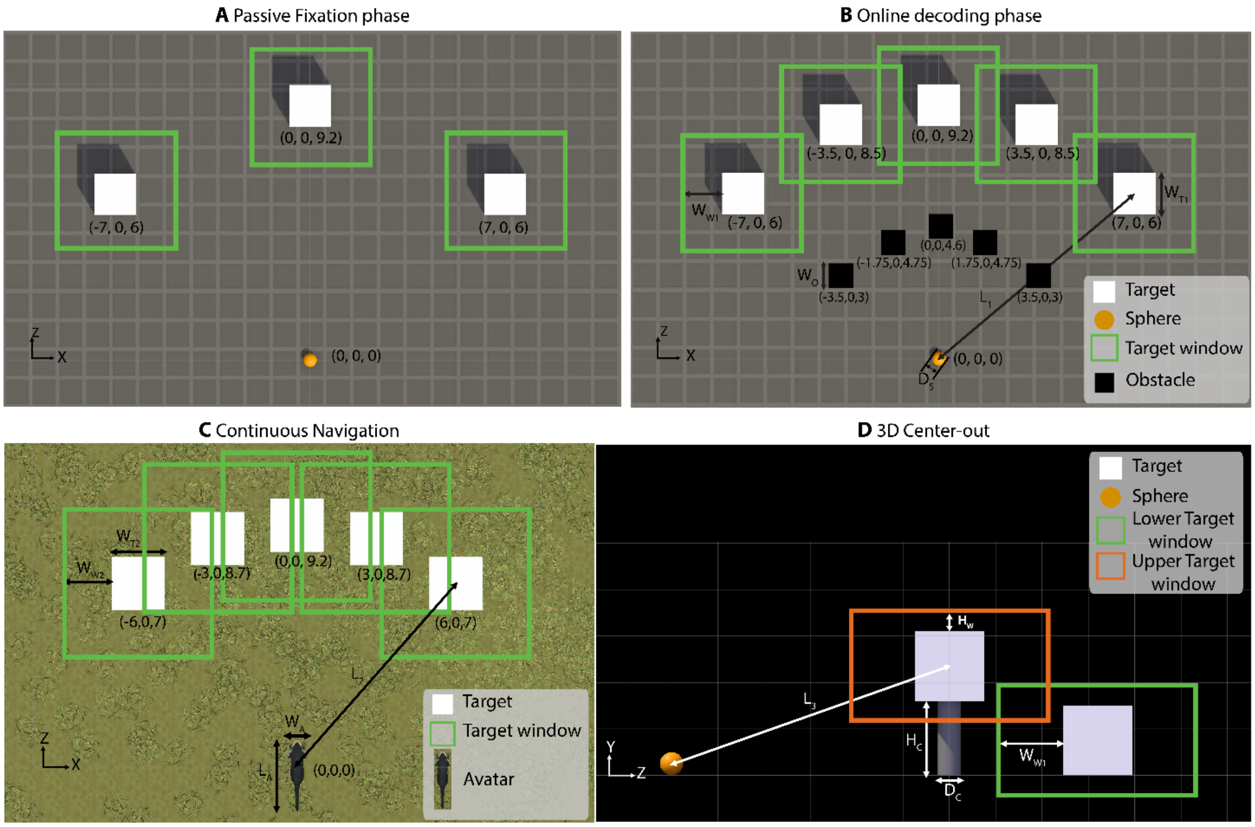

E

Measurement	Symbol	Length [u]
Diameter sphere	D_s	0.5
Length avatar	L_A	2.7
Width avatar	W_A	0.8
Height avatar	/	1.2
With target Center-out task	W_{T1}	1.5
Width target Continuous Navigation task	W_{T2}	2
Width obstacle	W_o	0.9
Sphere-target trajectory length Center-out task	L_1	~ 9.2
Avatar-target trajectory length Continuous Navigation task	L_2	~ 9.2
Sphere-target trajectory length 3D Center-out task	L_3	~ 9.2
Width target window Center-out task	W_{W1}	1.35
With target window Continuous Navigation task	W_{W2}	1.8
Height target window 3D Center-out task	H_W	0.45
Height cylinder	H_C	1.6
Diameter cylinder	D_C	0.3

Fig. S2. Dimensions, distances and coordinates of main and additional tasks. A Passive Fixation phase: only used 3 targets: left, straight and right. **B** Coordinates of sphere, targets and obstacles, together with different dimensions found Table **E,** for Center-out task, Obstacle and Respawn task. **C** Coordinates of sphere and targets, together with different dimensions found in Table **E**, for Continuous Navigation task. **D** Dimensions for 3D Center-out task, values found in Table **E**.

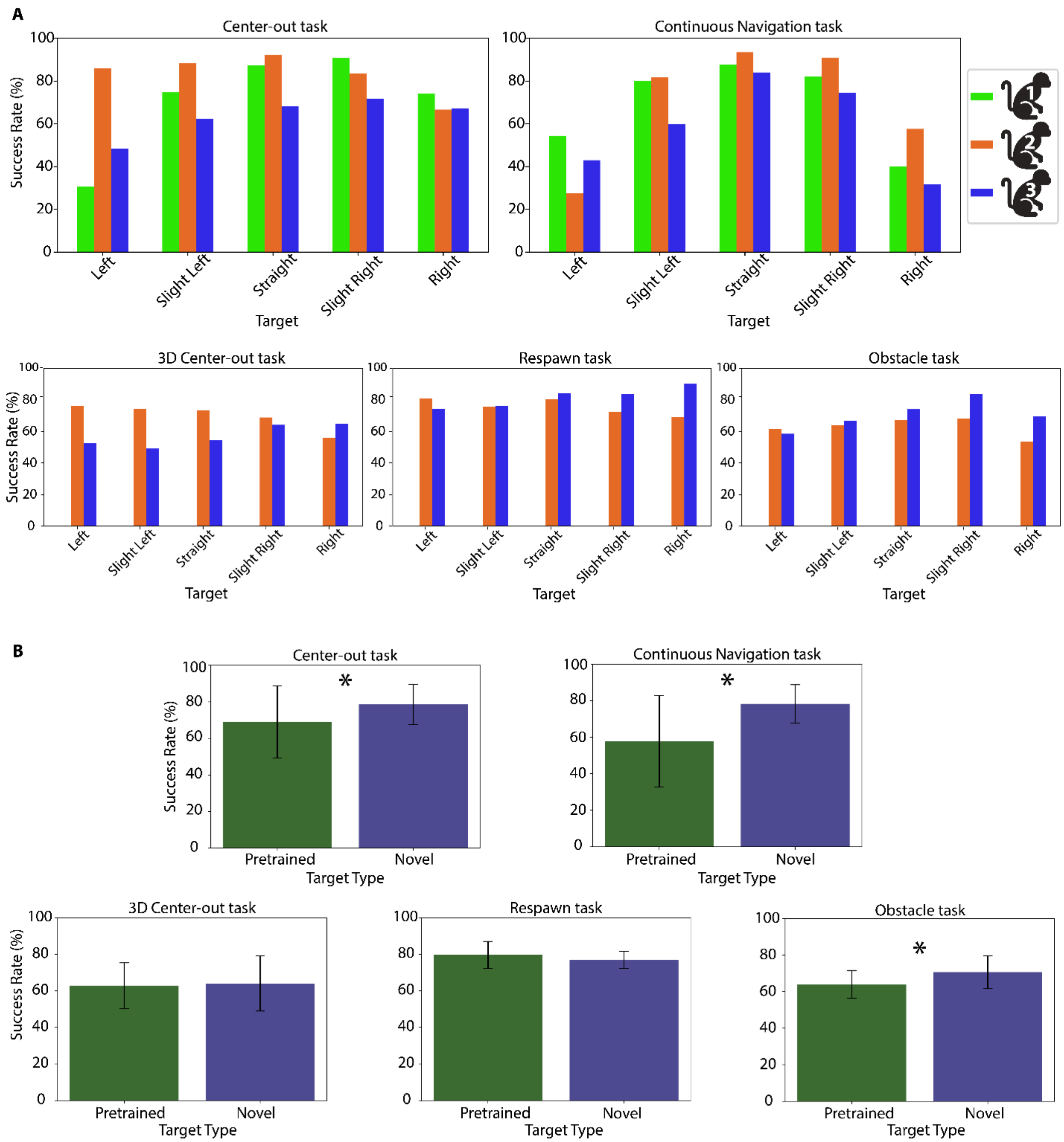

Fig. S3. Decoder generalization to novel targets across tasks. A Per-target success rate across tasks and monkeys. Each bar shows the mean ± standard deviation success rate per target position for individual monkeys for the different tasks. **B** Comparison of success rates for pretrained targets (left, straight, right) versus novel targets (slight left, slight right). Success rates were calculated per session and averaged across sessions. Statistical comparisons were performed using linear mixed-effects models with monkey identity as a random effect. Performance on novel targets significantly exceeded the performance on pretrained targets for Center-out, Continuous Navigation and Obstacle tasks ($p<0.05$). In the 3D Center-out and Respawn task there was no significant difference between novel and pretrained targets. The number of sessions (N) for each task is given in Table 1.

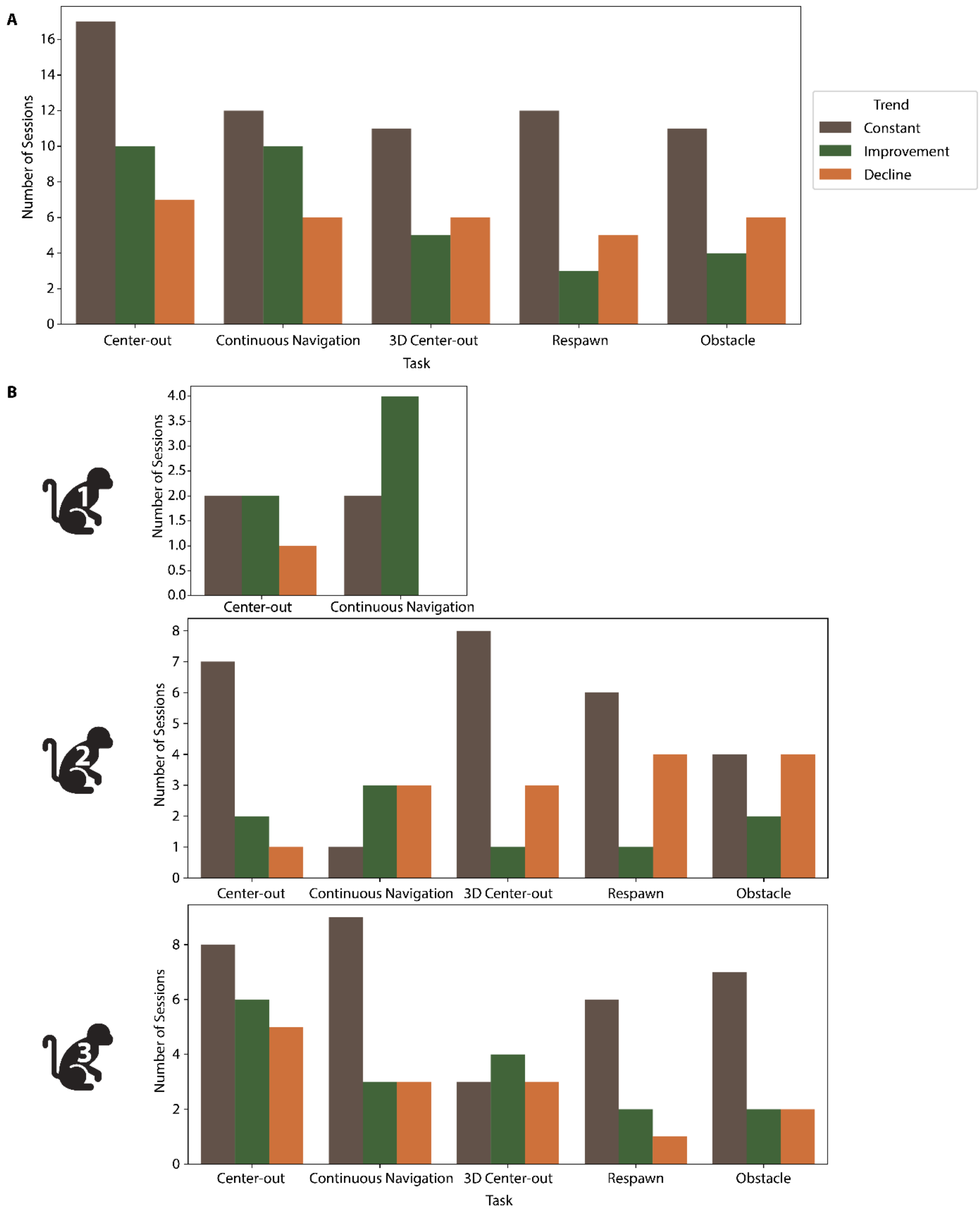

Fig. S4. Within-session performance trend analysis across tasks and monkeys. A Session-level trend classification (Improvement, Constant, Decline) across all sessions of each task, pooled across monkeys, based on linear fits to smoothed trial-by-trial success rates. Trends were classified by fitting a linear regression to smoothed trial-by-trial success rates and thresholding on slope sign and p-value (Improvement: $slope>0$, $p<0.05$; Decline: $slope<0$, $p<0.05$; Constant: $p\geq 0.05$). **B** Per-monkey breakdown of trend distributions across tasks. Monkeys differed in adaptation patterns: Monkey 1 showed the highest proportion of improvement and lowest decline, Monkey 2 had more decline sessions, and Monkey 3 showed intermediate behavior. The number of sessions (N) for each task can be found in Table 1.

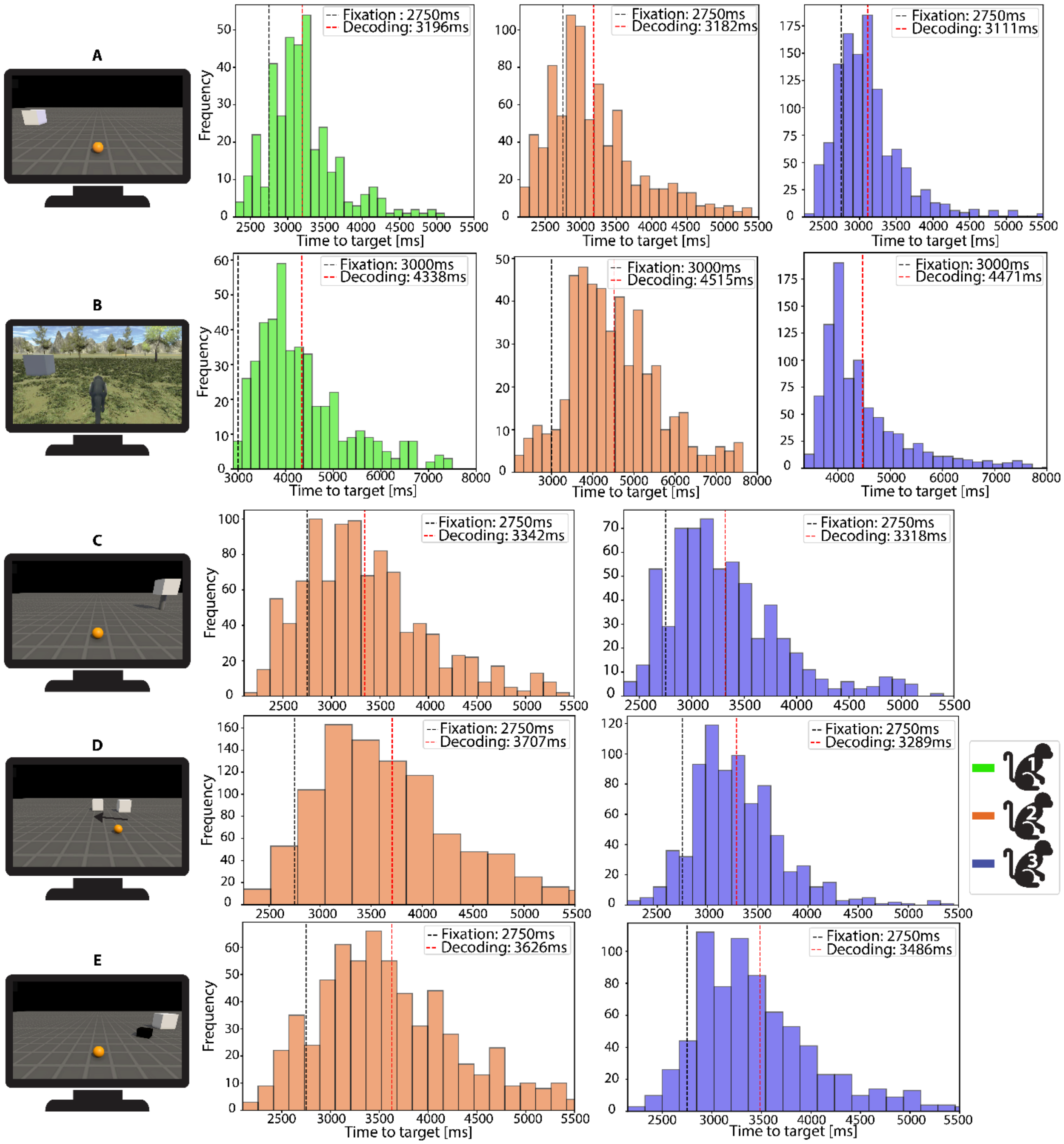

Fig. S5. Histogram of time to target for each trial during Online decoding phase and average time to target for Passive Fixation phase and Online decoding phase, for different tasks and monkey. A Center-out task. **B** Continuous Navigation task. **C** 3D Center-out task: time to target on average 21% slower in the Online Decoding phase (compared to the Passive Fixation phase). **D** Respawn task: average time to target in Online Decoding phase was 27% slower compared to the Passive Fixation phase. **E** Obstacle task: time to target was on average 29% slower compared to the Passive Fixation phase. For the Respawn and Obstacle tasks, it is important to note that the Passive Fixation phase did not include changes in target position or the presence of obstacles. The number of sessions (N) for each task is provided in Table 1.

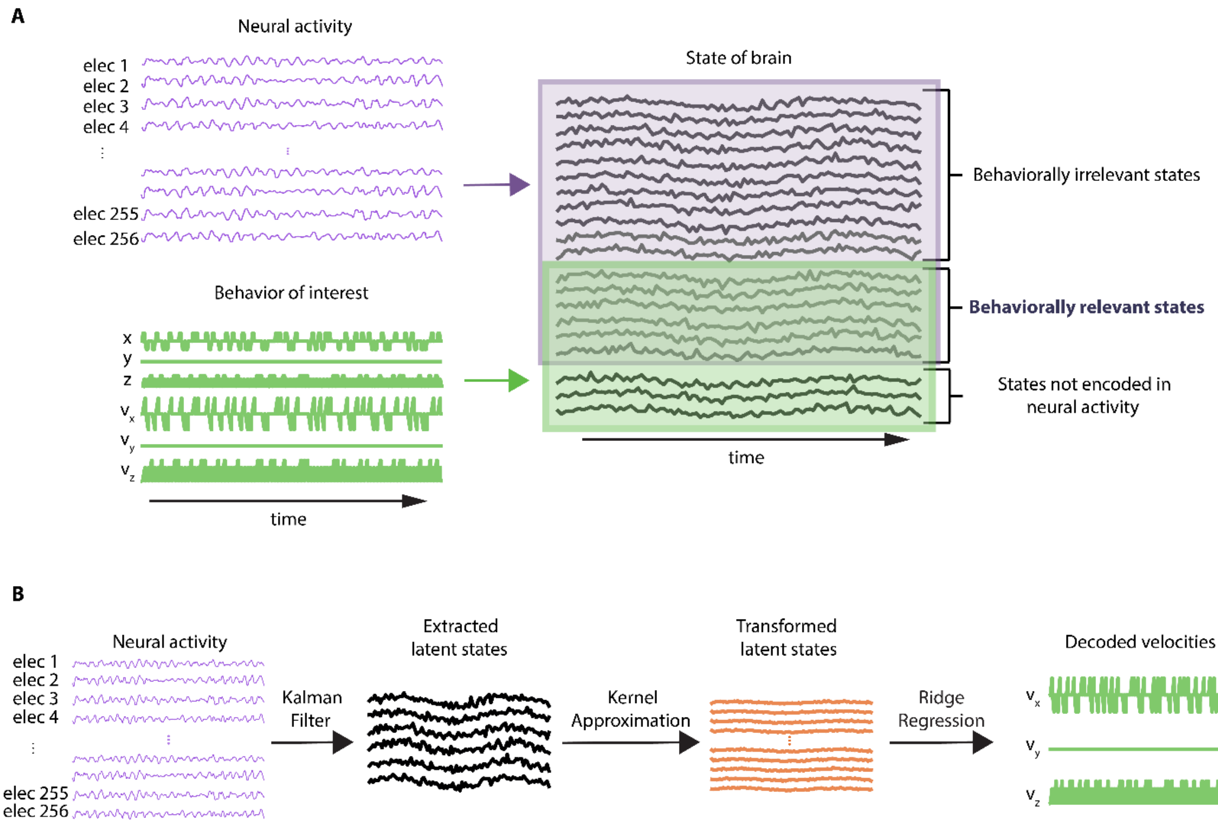

Fig. S6. Decoding algorithm scheme. A Extraction of behaviorally relevant states. Some dimensions of the latent variable (state of brain) may have driven the behavior of interest (velocity of sphere/avatar, green), some the neural activity (purple) and some both. The dimensions that drive both the neural activity and the behavior of interest are the behaviorally relevant states. Conceptual diagram inspired by (*41*), redrawn to illustrate the separation of latent state dimensions related to behavior and neural activity. **B** Computation of the decoded velocities: starting from the neural activity a Kalman filter was used to extract the behaviorally relevant latent states, then kernel approximation was used to transform these latent states to a higher dimensional space, lastly ridge regression was used to transform the latent states into velocities. These decoded velocities were used to control the sphere/avatar in real-time.

Monkey	Task	Test	Comparison	p-value
Monkey 1	Center-out	Binomial (1-sided)	PMd vs M1	3.13×10^{-2}
			PMv+PMd vs M1+PMd	3.13×10^{-2}
			PMd+PMd vs M1+PMv	3.13×10^{-2}
Monkey 1	Continuous navigation	Binomial (1-sided)	PMd vs M1	3.13×10^{-2}
			PMv vs M1	3.13×10^{-2}
			PMv+PMd vs M1+PMd	3.13×10^{-2}
Monkey 3	Center-out	Wilcoxon (2-sided)	PMd vs M1	7.7×10^{-3}
			PMv vs M1	4×10^{-6}
			PMv+PMd vs M1+PMd	1.57×10^{-2}
			PMv+PMd vs M1+PMv	4×10^{-6}

Table S1. Statistical comparisons of offline decoding performance across cortical area configurations. Summary of statistical tests comparing decoding success rates across cortical input configurations (see Fig. 2). Tests were performed separately for each monkey and task. For Monkey 1, one-sided exact binomial tests were used due to the limited number of sessions per condition. For Monkey 3, two-sided Wilcoxon signed-rank tests were used to account for repeated sessions. Only statistically significant comparisons are shown ($p < 0.05$). The number of sessions (N) for each task can be found in Table 1.

Monkey	Task	Velocity component	Correlation (mean±std)	Combined p-value
Monkey 1	Center-out	v	0.0 ± 0.01	0.65
		v_x	-0.02 ± 0.04	1.95×10^{-7}
		v_z	-0.01 ± 0.02	0.30
	Continuous Navigation	v	-0.01 ± 0.02	4.0×10^{-4}
		v_x	-0.04 ± 0.13	2.40×10^{-127}
		v_z	-0.02 ± 0.03	31.69×10^{-8}
Monkey 2	Center-out	v	0.11 ± 0.13	0.0
		v_x	-0.13 ± 0.1	0.0
		v_z	0.05 ± 0.13	2.16×10^{-198}
	Continuous Navigation	v	0.01 ± 0.09	1.87×10^{-57}
		v_x	0.02 ± 0.12	2.28×10^{-130}
		v_z	0.0 ± 0.08	3.41×10^{-29}
	3D Center-out	v	0.11 ± 0.11	3.25×10^{-304}
		v_x	-0.2 ± 0.11	0.0
		v_y	0.05 ± 0.11	1.43×10^{-196}
		v_z	-0.02 ± 0.1	3.23×10^{-132}
	Respawn	v	0.05 ± 0.09	9.64×10^{-146}
		v_x	-0.12 ± 0.13	0.0
		v_z	-0.06 ± 0.06	2.08×10^{-90}
	Obstacle	v	0.09 ± 0.05	1.49×10^{-90}
		v_x	-0.13 ± 0.12	4.82×10^{-293}
		v_z	-0.04 ±0.09	3.37×10^{-70}

Table S2. Spearman correlation between sEMG and movement velocity components across tasks. This table reports the average Spearman correlation coefficients (mean ± standard deviation) and combined p-values (Fisher's method) between surface EMG (sEMG) activity and each velocity component (v, v_x, v_y, v_z) for Monkey 1 and Monkey 2 across multiple tasks. All values reflect pooled session-level statistics for each condition. The number of sessions (N) for each task is provided in Table 1.

Monkey	Task	Velocity component	R^2 (median [IQR])		MSE (median [IQR])		Combine p-value	
			Linear	Non-linear	Linear	Non-linear	Linear	Non-linear
Monkey 1	Center-out	v	9.67 x 10^{-5} [1.15 x 10^{-4}]	1.64 x 10^{-3} [0.76 x 10^{-3}]	2.17 [0.2]	2.16 [0.2]	0.43	1.62 x 10^{5}
		v_x	6.67 x 10^{-4} [2.80 x 10^{-3}]	0.75 x 10^{-3} [3.90 x 10^{-3}]	2.07 [0.49]	2.06 [0.49]	1.17 x 10^{-11}	2.53 x 10^{-5}
		v_z	4.19 x 10^{-5} [7.61 x 10^{-4}]	1.84 x 10^{-3} [9.34 x 10^{-3}]	1.64 [0.04]	1.63 [0.04]	4.85 x 10^{3}	4.38 x 10^{6}
	Continuous Navigation	v	7.98 x 10^{-4} [1.57 x 10^{-3}]	1.05 x 10^{-3} [1.51 x 10^{-3}]	0.82 [0.23]	0.82 [023]	4.66 x 10^{-9}	1.48 x 10^{-2}
		v_x	6.0 x 10^{-3} [22.9 x 10^{-3}]	10.9 x 10^{-3} [25.6x 10^{-3}]	0.43 [0.06]	0.43 [0.06]	5.46 x 10^{-158}	2.44 x 10^{-42}
		v_z	1.15x 10^{-3} [2.47 x 10^{-3}]	1.52 x 10^{-3} [2.31 x 10^{-3}]	0.79 [0.2]	0.79 [0.2]	2.99 x 10^{-24}	0.09
Monkey 2	Center-out	v	9.47 x 10^{-3} [1.98 x 10^{-2}]	1.68 x 10^{-2} [2.59 x 10^{-2}]	1.13 [0.18]	112 [0.19]	1.27 x 10^{-150}	2.07 x 10^{-95}
		v_x	6.62 x 10^{-3} [2.81 x 10^{-2}]	1.69 x 10^{-2} [3.97x 10^{-2}]	2.64 [0.71]	2.59 [0.72]	0.0	2.54 x 10^{-105}
		v_z	5.37x 10^{-3} [9.44 x 10^{-3}]	8.30 x 10^{-3} [1.37 x 10^{-2}]	0.90 [0.21]	0.90 [0.21]	6.96 x 10^{72}	5.72 x 10^{51}
	Continuous Navigation	v	2.88 x 10^{-3} [6.83 x 10^{-3}]	6.23 x 10^{-3} [1.57 x 10^{-3}]	0.55 [0.59]	0.54 [0.59]	5.09 x 10^{-42}	5.38 x 10^{-13}
		v_x	8.32 x 10^{-3} [1.06 x 10^{-2}]	1.02 x 10^{-2} [1.34 x 10^{-2}]	1.23 [0.64]	1.22 [0.64]	1.77 x 10^{-129}	9.13 x 10^{-24}
		v_z	1.02 x 10^{-3} [2.49 x 10^{3}]	2.97 x 10^{-3} [3.12 x 10^{3}]	1.58 [0.65]	1.58 [0.64]	2.44 x 10^{-30}	4.44 x 10^{-11}
	3D Center-out	v	7.93 x 10^{-3} [1.65 x 10^{-2}]	1.25 x 10^{-2} [12.51 x 10^{-2}]	1.05 [0.39]	1.05 [0.39]	1.30 x 10^{-172}	4.98 x 10^{-98}
		v_x	3.56 x 10^{-2} [5.42 x 10^{-2}]	4.42 x 10^{-2} [4.34 x 10^{-2}]	2.78 [0.76]	2.74 [0.72]	0.0	4.52 x 10^{-184}
		v_y	1.09 x 10^{2} [2.45 x 10^{-2}]	1.45 x 10^{2} [2.27 x 10^{-2}]	0.06 [0.02]	0.06 [0.02]	6.81 x 10^{-220}	2.29 x 10^{-62}
		v_z	4.55 x 10^{-3} [6.30 x 10^{-3}]	5.50 x 10^{-3} [1.26 x 10^{-2}]	0.74 [0.36]	0.73 [0.36]	1.81 x 10^{-102}	9.70 x 10^{-43}
	Respawn	v	6.59 x 10^{-3} [1.95 x 10^{-2}]	8.44 x 10^{-3} [1.64 x 10^{-2}]	0.83 [0.24]	0.82 [0.24]	7.74 x 10^{-181}	3.03 x 10^{-21}
		v_x	3.08 x 10^{-2} [8.41 x 10^{-2}]	5.45 x 10^{-2} [7.97x 10^{-2}]	2.26 [0.76]	2.26 [0.76]	0.0	1.48 x 10^{-115}
		v_z	1.70x 10^{-3} [1.04 x 10^{-2}]	2.58x 10^{-3} [1.72 x 10^{-2}]	0.70 [0.06]	0.70 [0.067]	1.02 x 10^{-111}	1.00 x 10^{-36}
	Obstacle	v	6.12 x 10^{-3} [9.57 x 10^{-3}]	9.91 x 10^{-3} [8.34x 10^{-3}]	1.08 [0.26]	1.08 [02.6]	1.33 x 10^{-68}	6.57 x 10^{-19}
		v_x	1.60 x 10^{-2} [2.22 x 10^{-2}]	3.13 x 10^{-2} [4.32 x 10^{-2}]	2.58 [0.30]	2.57 [0.32]	2.07 x 10^{-206}	1.88 x 10^{-133}
		v_z	2.22 x 10^{-3} [3.36 x 10^{-3}]	3.36 x 10^{-3} [4.48 x 10^{-3}]	0.85 [0.10]	0.85 [0.10]	6.17 x 10^{-42}	5.02 x 10^{-7}

Table S3. Regression performance metrics between sEMG and movement velocity components. This table summarizes the results of both linear and non-linear regression analyses relating sEMG signals to different velocity components (v, v_x, v_y, v_z) for Monkey 1 and Monkey 2 across multiple tasks. Reported metrics include the median R^2 with interquartile range (IQR), median MSE with IQR, and the combined p-value (computed using Fisher's method) across sessions. Results are shown separately for linear and non-linear models. The number of sessions (N) for each condition is reported in Table 1.

Monkey	Task	Velocity component	Correlation (mean±std)	Combined p-value
Monkey 1	Center-out	v	0.008 ± 0.09	2.04×10^{-32}
		v_x	0.56 ± 0.05	0.0
		v_z	-0.11± 0.03	1.71×10^{-57}
	Continuous Navigation	v	-0.01 ± 0.03	1.54×10^{-12}
		v_x	0.27 ± 0.10	0.0
		v_z	-0.02 ± 0.04	3.91×10^{-16}
Monkey 2	Center-out	v	-0.17 ± 0.13	0.0
		v_x	0.75 ± 0.04	0.0
		v_z	-0.12 ± 0.10	3.55×10^{-299}
	Continuous Navigation	v	0.02 ± 0.10	4.03×10^{-60}
		v_x	0.07 ± 0.07	2.22×10^{-76}
		v_z	-0.02 ± 0.08	3.97×10^{-60}
	3D Center-out	v	-0.16 ± 0.14	0.0
		v_x	0.73± 0.06	0.0
		v_y	-0.09 ± 0.16	0.0
		v_z	-0.12 ± 0.09	2.04×10^{-285}
	Respawn	v	-0.07 ± 0.21	0.0
		v_x	0.67 ± 0.06	0.0
		v_z	-0.07 ± 0.14	0.0
	Obstacle	v	-0.03 ± 0.17	8.77×10^{-260}
		v_x	0.67 ± 0.07	0.0
		v_z	-0.04 ±0.16	2.15×10^{-230}
Monkey 3	Center-out	v	0.02 ± 0.09	3.43×10^{-113}
		v_x	0.70 ± 0.06	0.0
		v_z	-0.09 ± 0.09	3.14×10^{-233}
	Continuous Navigation	v	-0.05 ± 0.09	1.17×10^{-143}
		v_x	0.02 ± 0.08	9.05×10^{-76}
		v_z	-0.008 ± 0.05	4.65×10^{-34}
	3D Center-out	v	0.01 ± 0.12	5.83×10^{-104}
		v_x	0.60 ± 0.09	0.0
		v_y	-0.007 ± 0.21	42.9×10^{-282}
		v_z	-0.09 ± 0.12	3.32×10^{-156}
	Respawn	v	0.07 ± 0.08	1.62×10^{-115}
		v_x	0.61 ± 0.07	0.0
		v_z	-0.02 ± 0.09	4.82×10^{-82}
	Obstacle	v	0.10 ± 0.10	3.37×10^{-199}
		v_x	0.64 ± 0.08	0.0
		v_z	0.03 ±0.12	2.85×10^{-124}

Table S4. Spearman correlation and combined p-value between x-component of eye movement and different velocity components for Monkey 1, 2 and 3, for various tasks. The mean ± standard deviation Spearman correlation and combined p-value (Fisher's method) across all sessions. N (number of sessions) is listed in Table 1.

A

Study	Task	Species	Brain area	Environment	Training phase	Decoder	Recalibration	Feedback	Overt movements
Serruya et al. (2002)	2D cursor control	Monkey	M1	2D screen	Arm movement	Linear decoder (linear)	No	Visual + proprioception	Training: Yes<br>Decoding: Yes
Taylor et al. (2002)	3D cursor control	Monkey	M1	2D screen	Arm movement	Population vector algorithm (linear)	No	Visual + proprioception	Training: Yes<br>Decoding: Yes
Gilja et al. (2012)	2D cursor control	Monkey	PMd + M1	2D screen	1. Arm movement<br>2. Retraining	Kalman Filter + ReFIT (linear)	No	Visual + proprioception	Training: Yes<br>Decoding: Yes
Jarosiewicz et al. (2015)	2D cursor control for typing	Human	M1	2D screen	Passive observation + attempted movement	Self calibrating Kalman filter (Adaptive Linear)	Yes	Visual	Training: No<br>Decoding: No<br>Residuall: minimal EMG
Pandarinath et al. (2017)	2D cursor control for typing	Human	M1	2D screen	1. Passive observation + attempted movement<br>2. Assisted online decoding	Kalman filter + ReFIT (linear)	No	Visual	Training: No<br>Decoding: No<br>Residual: No EMG
Rajangam et al. (2016)	Wheelchair navigation	Monkey	PMd + M1	Real environment	Passive motion	Wiener filter (linear)	No	Visual + vestibular + proprioception	Training: Yes<br>Decoding: Yes
Schroeder et al. (2022)	1D cycling motion	Monkey	PMd + M1	2D screen (virtual corridor)	Manual cycling with arm movement	Cycling subspace state-machine decoder	No	Visual	Training: Yes<br>Decoding: Yes<br>Residual: No EMG
Hochberg et al. (2006)	2D cursor + 3D endpoint control of robotic arm	Human	M1	Virtual robotic arm	Passive observation + attempted movement	Linear decoder (linear)	No	Visual	Training: No<br>Decoding: No<br>Residual: No EMG
Hochberg et al. (2012)	3D endpoint control of robotic arm	Human	M1	Robotic arm	Passive observation + attempted movement of 2D cursor	Kalman filter (linear)	Yes	Visual	Training: No<br>Decoding: No<br>Residual: No EMG
Collinger et al. (2013)	3D endpoint control of robotic arm (+wrist rotation + grasp)	Human	M1	Robotic arm	1. Passive observation + attempted movement of robot<br>2. Assisted online decoding	Optimal Linear Estimation (linear)	No	Visual	Training: No<br>Decoding: No<br>Residual: No EMG
Wodlinger at al. (2015)	3D endpoint control of robotic arm (+ wrist rotation + hand shape)	Human	M1	Robotic arm	1. Passive observation + attempted movement of robot<br>2. Assisted online decoding	Optimal Linear Estimation (linear)	No	Visual	Training: No<br>Decoding: No<br>Residual: No EMG
Willsey et al. (2025)	2D Finger velocity	Human	M1	Hand on 2D screen	1. Attempted movement<br>2. Retraining	RNN (linear) + ReFIT	No	Visual	Training: No<br>Decoding: No<br>Residual: No EMG
**Current Study**	**2D & 3D Navigation + obstacle avoidance + change in intention**	**Monkey**	**PMv PMd M1**	**Virtual reality**	**Passive observation on screen**	**Non-linear extension of PSID (non linear)**	**No**	**Visual**	**Training: No<br>Decoding: No<br>Residual: No**

B

Study	Task	Species	Brain area	Environment	Training phase	Decoder	Recalibration	Feedback	Overt movements
Santhanam et al. (2006)	2D cursor control: target selection	Monkey	PMd	2D screen	Arm movement	Kalman filter + optimal stopping	No	Visual + proprioception	Training: Yes<br>Decoding: Yes
Carmena et al. (2003)	2D cursor control: target selection	Monkey	M1<br>PMd	Robotic arm	Arm movement + joystick	Linear decoder	No	Visual + proprioception	Training: Yes<br>Decoding: Yes
Velliste et al. (2008)	3D prosthetic control: position decoding	Monkey	M1	Robotic arm	Arm movement + joystick	Linear decoder	No	Visual + proprioception	Training: Yes<br>Decoding: Yes
Libedinsky et al. (2016)	Wheelchair navigation 4-class classification	Monkey	M1	Real-environment	1. Joystick<br>2. Assisted decoder training	LDA classifier	No	Visual + vestibular + proprioception	Training: Yes<br>Decoding: Yes
Willett et al. (2021)	Imagined handwriting (classification)	Human	M1	2D screen	Attempted handwritting	Recurrent Neural Network (RNN)	No	Visual	Training: No<br>Decoding: No<br>Residual: No EMG
Willet et al. (2023)	Speech decoding	Human	Broca's area<br>PMv	Auditory/ speech interface	Attempted speech	RNN-based classifier	No	Visual + auditory	Training: Yes<br>Decoding: Yes<br>Residual: No EMG

Table S5. Intracortical motor BCI studies using velocity, position, classification, or language decoding. A Summary of representative intracortical BCI studies that employ continuous velocity decoding in motor tasks with online feedback. For each study, we report task type, species, environment, training paradigm, decoder architecture, recalibration, feedback modality, and the presence of overt movement or EMG monitoring. Our current study (highlighted) is the only one to feature nonlinear decoding of 3D navigation using passive observation alone, without decoder recalibration or overt movement. Success rates and performance metrics vary across studies and are not

directly comparable due to differences in task design, autonomy levels, and success criteria. Key performance indicators are provided below for contextual understanding: Serruya (2002): not reported; Taylor (2004): 49%; Gilja (2012): 100% (target window enlarged for method comparison); Hochberg (2006): 73–95%; Hochberg (2012): 69–96%; Collinger (2013): 85% (7 DOF); Wodlinger (2015): 70% (10 DOF); Jarosiewicz (2015): typing – 12 characters/min; Pandarinath (2016): typing – 12–39 characters/min. **B** Complementary studies using position decoding, classification, or language models rather than continuous velocity decoding. While not directly comparable, these studies illustrate the diversity of intracortical decoding strategies across motor, cognitive, and speech-related tasks.

Movie S1.

Online Decoding phase Center-out task – Monkey 2.

Movie S2.

Online Decoding phase Continuous Navigation task – Monkey 1.

Movie S3.

Online Decoding phase Continuous Navigation first person perspective task – Monkey 3.

Movie S4.

Online Decoding phase 3D Center-out task – Monkey 2.

Movie S5.

Online Decoding phase Respawn task – Monkey 2.

Movie S6.

Online Decoding phase Obstacle task – Monkey 3.

Movie S7.

Passive Fixation phase Continuous Navigation task.
